# The teaching of pediatric emergency procedures to medical students - a pilot study

**DOI:** 10.1186/1757-7241-23-S1-A48

**Published:** 2015-07-16

**Authors:** Camilla B Sørensen, Sandra R Viggers

**Affiliations:** 1 Students' Society for Anaesthesiology and Traumatology, Faculty of Health Sciences, Copenhagen University, Copenhagen, Denmark

## Background

Junior doctors are often first on scene in the emergency department. It is therefore crucial that they are capable of performing life-saving interventions (LSI's). Pediatricians and anesthesiologists handle pediatric emergencies. However, depending on hospital location, local structure, and resources junior doctors can be compelled to perform these LSI's. Many junior doctors will not learn additional pediatric skills after the end of the assigned curriculum in medical school. We designed a four-hour workshop-based course to introduce Danish medical students to clinical skills needed to manage pediatric emergencies.

## Methods

A total of 12 medical students all in their fourth-sixth year of medical school participated in a four-hour course in pediatric procedures. Students were divided into groups of four. The course included workshops with theory and practical training in intraosseous access (IO), foreign body airway obstruction (FBAO), neonatal resuscitation (NR), and umbilical vein catheterization (UVC). Prior to the workshops the students were presented with a multiple-choice questionnaire (MCQ). The MCQ was repeated at the end of the course. At the end of the training sessions an objective structured clinical examination (OSCE) was performed and the students were assessed using a graded (0-3 points) standardized checklist. Data from the MCQ tests were analyzed using the Wilcoxon Signed Rank test and a p-value < 0.01 was considered significant.

## Results

A total of 12 medical students performed the pre and post MCQ test and the OSCE. The MCQ test shows a significant improvement in the post-test (W = 78 Z = 3.04, p < 0.005). For UVC the mean score in the OSCE was 7.67 (3-9). The maximum obtainable score for the whole group was 108 points. The participants scored a total of 85% (92/108). For FBAO, mean = 9.25 (6-10) and total OSCE score = 93% (111/120). For NR, mean = 9.17 (6-11) and total OSCE score = 83% (110/132). For I.O, mean = 8.42 (7-9) and total OSCE score = 93% (100/108).

## Conclusion

This small pilot study indicates that students can learn to perform pediatric LSIs, at least in an educational environment. The students performed well in the MCQ but OSCE identified some gaps in performance. Future research in students' ability to translate theoretical knowledge into practical skills is necessary.

